# Supplementary material for: Natural Foraging Selection and Gut Microecology of Two Subterranean Rodents from the Eurasian Steppe in China
Source: Animals (Basel). 2024 Aug 13;14(16):2334. doi: 10.3390/ani14162334 (PMC11350848; doi:10.3390/ani14162334)

Supplemental Information for:

Natural foraging selection and gut microecology of two subterranean rodents from the Eurasian Steppe in China

Zhenghaoni Shang[1,2,3], Kai Chen[1,2,3], Tingting Han[1,2,3], Fan Bu[1,2,3], Shanshan Sun[1,2,3],

Na Zhu[1,2,3], Duhu Man[4], Ke Yang[5], Shuai Yuan [1,2,3*], Heping Fu [1,2,3*]

[1] College of Grassland Resources and Environment, Inner Mongolia Agricultural University, Hohhot 010011, China; shangzhn1997@163.com (Z.S.); ckai1414@163.com (K.C.); hantingting1015@163.com (T.H.); bufanimau@163.com (F.B.); sunshanshan557@163.com (S.S.); jvn0397@163.com (N.Z.)

[2] Key Laboratory of Grassland Rodent Ecology and Pest Controlled, Hohhot 010011, China

[3] Key Laboratory of Grassland Resources, Ministry of Education, Hohhot 010011, China

[4] College of Agriculture, Hulunbuir University, Hulunbuir 021000, China; mantou08@126.com

[5] Alxa League Meteorological Bureau, Alxa 750300, China; yangke19961006@163.com

* Correspondence: yuanshuai2020@163.com (S.Y.); fuheping@126.com (H.F.)

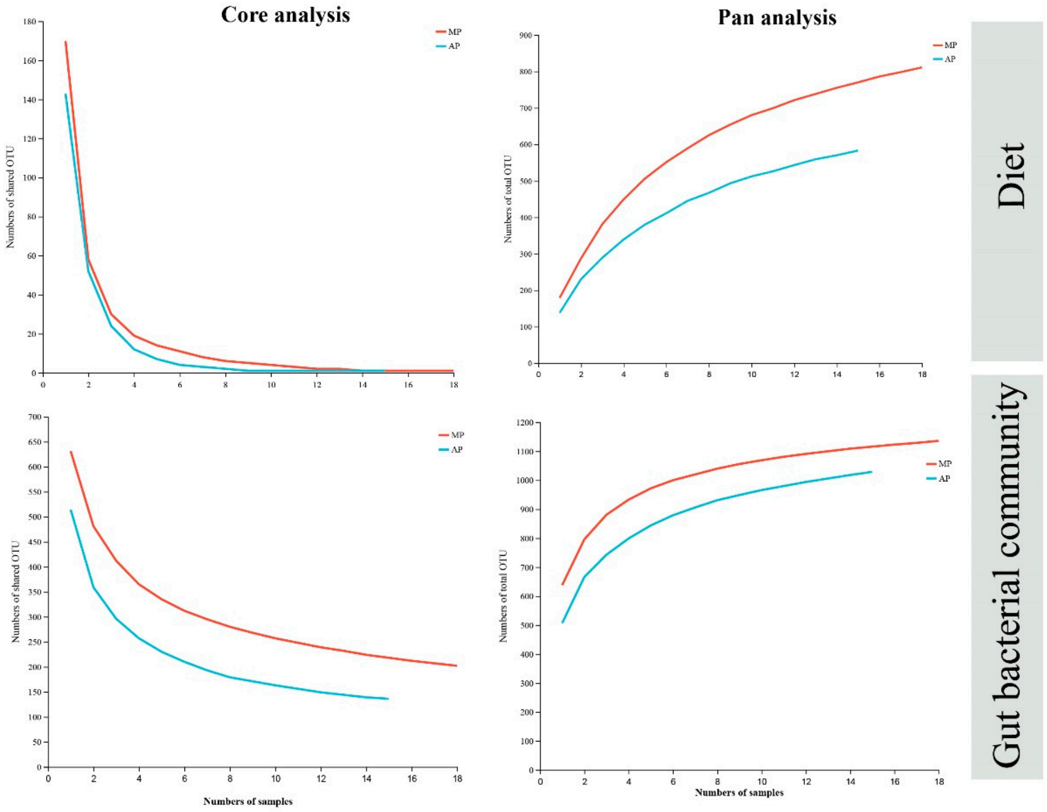

Supplementary Figure S1: Pan and Core OTU analysis

Supplementary Table S1: Food composition of two kinds of zokor

Group	*M. psilurus* only	Both	*M. aspalax* only
Species	*Bistorta officinalis*	*Allium cepa*	*Nicotiana tabacum*
	Bromus pumpellianus	*Allium tuberosum*	*Potentilla acaulis*
	Bupleurum longiradiatum	*Artemisia stolonifera*	*Potentilla chinensis*
	Caragana arborescens	*Bupleurum scorzonerifolium*	*Zea mays*
	Chrysanthemum indicum	*Elymus dahuricus*	
	Coreopsis tinctoria	*Clematis aethusifolia*	
	Galium dahuricum	*Rubus idaeus*	
	Galium pogonanthum	*Sanguisorba officinalis*	
	Helictochloa hookeri	*Saussurea japonica*	
	Hordeum vulgare	*Synurus deltoides*	
	Iris bungei	*Thalictrum minus*	
	Iris uniflora	*Thalictrum squamiferum*	
	Koenigia davisiae	*Trisetum spicatum*	
	Lespedeza bicolor		
	Linaria vulgaris		
	Paeonia lactiflora		
	Patrinia scabra		
	Phedimus aizoon		
	Phedimus kamtschaticus		
	Poa annua		
	Pulsatilla cernua		
	Rumex crispus		
	Silene repens		
	Viola phalacrocarpa		
	Youngia japonica		

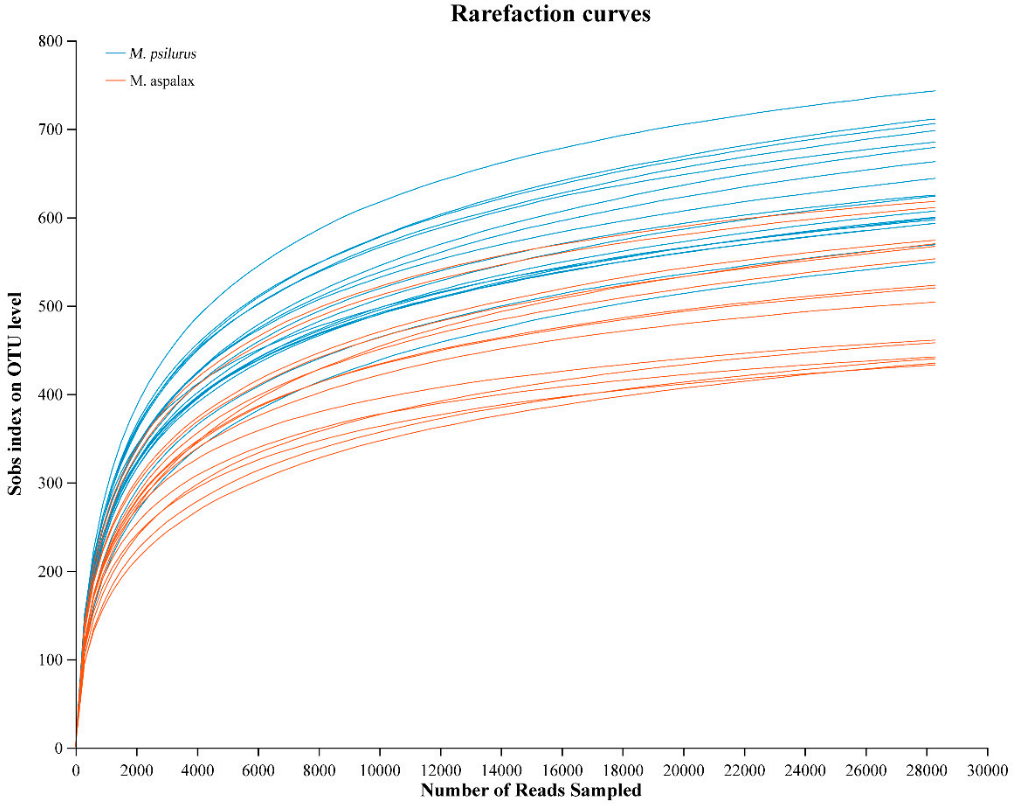

Supplementary Figure S2: Rarefaction curves for all analyzed samples

Supplementary Figure S3: Gut microbial communities of two species of zokors at the genus level

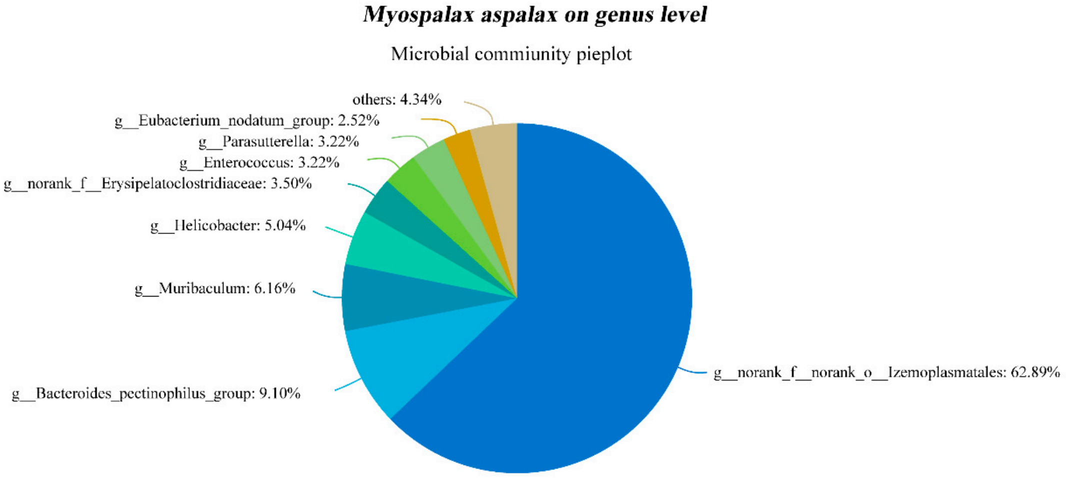

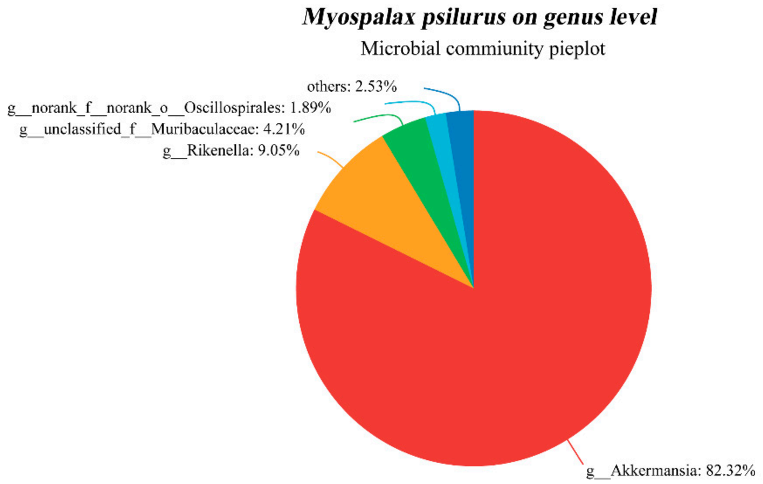

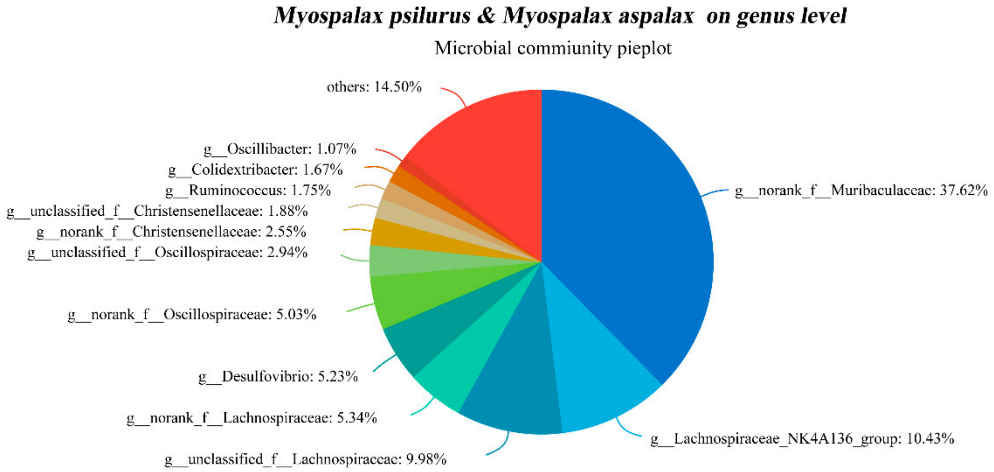

Supplement: Supplementary file 1 [file animals-14-02334-s001.zip › animals-3099120-supplementary.pdf]
